# The discovery of autism: Indian parents’ experiences of caring for their child with an autism spectrum disorder

**DOI:** 10.1177/1363461512447139

**Published:** 2012-07

**Authors:** Miraj U. Desai, Gauri Divan, Frederick J. Wertz, Vikram Patel

**Affiliations:** Fordham University and Sangath, Goa, India; Sangath, Goa, India; Fordham University; London School of Hygiene & Tropical Medicine and Sangath, Goa, India

**Keywords:** autism spectrum disorders, qualitative research, phenomenological methods, India, low- and middle-income countries

## Abstract

The current study investigated the lived experience of 12 parents of children with an Autism Spectrum Disorder in everyday cultural contexts in Goa, India. Narratives from parents collected between 2009 and 2010 were analyzed using the procedures of phenomenological psychology. Four temporal phases of parents’ experience emerged from these data. Findings showed that the earliest phase of the child’s life was a period of relative normalcy and social cohesion. In the second phase, the child’s behaviors began to disrupt the everyday social order, but parents viewed these unexpected behaviors as temporary. In the third phase, parents’ observations in public situations, along with assessments of others, led to a qualitative shift in which parents began to perceive that there was a persisting problem interfering with their child’s social and practical activities. In the fourth phase, parents grappled with developing their child’s capacities to meet existing practical opportunities in the local society, while attempting to reshape the social world to accommodate the abilities and limits of children like their own. Parents’ fundamental concerns throughout their journey were: learning to meet new and unfamiliar challenges as parents, caring for their child’s basic needs, and finding an engaging niche with a sense of belonging for their child in the everyday milieu. Both culture-specific and potentially universal levels of experience are delineated in the overall findings. Implications for culturally sensitive research and practice in India and other low- and middle-income countries are discussed.

Since Kanner (1943) first identified a group of children with “autistic disturbances of affective contact” in the United States (1943, p. 217), classic autism and the broader group autism spectrum disorders (ASD) have been formally adopted by various professional diagnostic systems (e.g., Diagnostic and Statistical Manual of Mental Disorders [DSM-IV-TR; American Psychiatric Association, 2000]; International Classification of Diseases [ICD-10; World Health Organization, 1992]). These diagnostic constructs have since “traveled around the globe,” as evidenced by the existence of national organizations for autism in over 80 countries (Daley, 2002, p. 532). With respect to India in particular, knowledge of Western psychiatry and psychology first arrived with British colonialism (Daley, 2004). A “child showing schizophrenic behavior” was first mentioned in 1959 (Batliwalla, 1959, p. 251), and autism-specific research has substantially increased since then (Daley, 2004). Sustained professional focus on autism began in the late 1980s and 1990s (Krishnamurthy, 2008), and autism is now widely recognized in medical, political, and legal circles in India (Daley, 2004; Daley & Sigman, 2002). The current study contributes to the extant literature by providing an in-depth, qualitative investigation of parenting a child with ASD in the Indian state of Goa.

Kleinman (1977) suggested that “ideal” cross-cultural studies begin with “local phenomenological descriptions” (1977, p. 4). Instead of presupposing the nature of psychopathology from a culturally distant perspective, local descriptions are required to provide an understanding of it in cultural contexts. Knowledge of local perspectives has helped strengthen community-based care and improve outcomes for individuals experiencing significant psychological distress (e.g., Chatterjee et al., 2008). In the case of autism, evidence from countries such as the USA (Altiere & von Kluge, 2009), Israel (Shaked, 2005), the UK (Avdi, Griffin, & Brough, 2000b), and Australia (Gray, 1993, 1995, 2001), for instance, has shown that parent perspectives often differ from traditional scientific/professional ones and frequently invoke spiritual, moral, or personal interpretations. Because parents have been shown to find meaning by using nonmedical or nonprofessional interpretive frameworks and regularly turn to alternative treatments for ASD, an investigation of what matters to parents on an everyday level in other cultures is also necessary. In line with this general trend of exploring indigenous viewpoints, professional organizations such as the American Psychological Association Presidential Task Force on Evidence-Based Practice (2006) have recently stated that an understanding of patients’ culture, preferences, values, and worldview is an integral part of evidence-based practice in psychology (EBPP).

In India, little research has sought to examine parents’ indigenous viewpoints of their children diagnosed with ASD. The available literature has suggested that children with ASD often do present families with considerable challenges requiring external support (Daley, 2004; Gupta & Singhal, 2005; Kishore & Basu, 2011; Krishnamurthy, 2008; Malhotra, Chakrabarti, Gupta, & Gill, 2004). Daley’s (2004) research in particular offered several insights into Indian parents’ perspectives on ASD. For instance, Daley found that Indian parents’ initial symptom recognition in children who were eventually diagnosed with ASD was at a range of 6 to 10 months later than families in the West. Potential reasons given for the extra time in India prior to help-seeking included local cultural beliefs like “boys speak late,” which perhaps normalized early unusual behaviors. Overall, the initial symptoms most reported were social difficulties, suggesting that a child’s ability to relate to others is of high import to parents in India. More recent research in India on parental perceptions of intellectual disabilities has also noted diverse frames of reference featuring sociocultural, spiritual, and biomedical components (Edwardraj, Mumtaj, Prasad, Kuruvilla, & Jacob, 2010).

The extant literature provides some knowledge of the influence of Indian cultural beliefs and social norms on the local responses to ASD. However, more information is needed on parents’ indigenous viewpoints, prior to theoretical conceptualizations like symptoms and diagnosis. Parents may not initially be using these latter constructs to understand and describe their child, so it is important to examine the full meaning of their original perspectives and how these may change over time. In addition, research conducted in a different Indian location than previous research, with both urban and rural populations, may allow for knowledge of how parenting concerns and practices may be common or different across various settings (see Grinker, 2007). The phenomenological approach was utilized in order to freshly access and concretely describe the parents’ lived experiences of their child with sensitivity to local context and meaning. The research was embedded in a larger project on ASD in India (Autism Research and Training Initiative: ARTI) based at a community-based, nongovernmental organization (NGO) and child development center located in Goa, India.

## Method

### Setting

The study was conducted in the Indian state of Goa. Goa differs from other parts of India given its Portuguese colonial heritage and relatively high educational and economic development. The state has a population of 1.4 million and is characterized by geographical, cultural, and religious diversity. The most commonly spoken language is Konkani, but Marathi, English, and Hindi are also used. The overall Autism Research and Training Initiative (ARTI) project investigated several domain areas related to ASD in India, including help-seeking patterns and family impact. The current study focused on parents’ experiences of and with their child from birth to the present time. Formal Institutional Review Board approval was granted by Sangath Centre and the London School of Hygiene and Tropical Medicine prior to the study.

### Participants

Parents were recruited via key informants at special schools, child development centers, and health clinics. Primary inclusion criteria were parents with a child who was already identified as meeting criteria for ASD. Diagnoses had been given by pediatricians, local clinical psychologists, and special educators using standard DSM-based criteria.

The sample for the present study consisted of 12 parents of 10 children: one father and seven mothers were interviewed alone (i.e., eight parents of children with ASD); and two sets of parents, mother and father, were interviewed together (i.e., four parents of children with ASD). The sample size was consistent with standards for phenomenological research (Giorgi, 2009; Starks & Trinidad, 2007). The participants were deliberately selected to reflect key differences in the local population, such as religious and geographical background. Table 1 displays information on these demographic characteristics and others including family type (nuclear/joint). Joint families are those with more than one “immediate” family living in one household and often include additional extended family members.

**Table 1.** Participant demographics

Participants: (M)other; (F)ather	Parental age (M)other; (F)ather	Religion	Community type	Family type	Gender of child	Age of child	Age of child at diagnosis
M1 F1	M1 49 F1 NA	Hindu	Urban	Joint	Female	23	4.5
M2 F2	F2 37 M2 32	Hindu	Urban	Joint	Male	6	3
F3	F3 53	Hindu	Rural	Joint	Male	8	8
M4	M4 32	Hindu	Rural	Joint	Male	12	7
M5	M5 48	Catholic	Urban	Nuclear	Male	16	8
M6	M6 40	Catholic	Urban	Nuclear	Male	12	5
M7	M7 38	Hindu	Rural	Joint	Male	7	1.5
M8	M8 30	Hindu	Urban	Joint	Male	8	3
M9	M9 N/A	Hindu	Urban	Joint	Male	7	3.5
M10	M10 39	Catholic	Rural	Joint	Male	5	1.5

### Data collection

The data for the study were comprised of in-depth interviews (Davidson, 2003; Giorgi, 2009; Kvale, 2008) conducted by members of ARTI between June 2009 and August 2010. Interview staff consisted of one doctoral-, two master’s-, and three bachelor-level personnel, conducting interviews in teams of two (one interviewer, one note-taker). All interviewers were trained and supervised by doctoral-level senior researchers with extensive experience in qualitative research. Interviews were completed in various settings, such as participants’ homes, child clinics, and special schools. The team made every effort to accommodate participants’ location preferences. For example, some parents preferred the school setting and time because they could participate in the research while waiting for their child, whereas others preferred the more familiar home environment. Interviews in formal settings such as schools were conducted in private rooms to minimize distractions and increase comfort. All participants gave consent for interviews and a separate consent for audio recording. Participants were assured that their participation was voluntary and confidential.

Each interview was conducted in the language of the participants’ preference (Konkani, Marathi, English, or Hindi) and lasted 1–3 hours, with later follow-up questions as needed. The present study utilized a semistructured interview guide, which elicited in-depth narratives from parents of life with the child from birth to the present (see Appendix). Interviewers remained flexible and open to topics introduced by participants, who guided the researcher through their particular narratives of parenting a child identified as ASD. Key events explored were: birth and early experiences with the child; parents’ first notice of behavioral differences; and interactions with family members, healthcare providers, educators, and community members in relation to their child. Throughout the process, completed interviews were examined by senior researchers to monitor the data quality and to provide feedback to the interviewers.

### Data analysis

The tape-recorded interviews were transcribed into electronic format. Konkani, Marathi, or Hindi interviews were translated directly into English by research members fluent in both languages. Redundancy that offered no added meaning was removed. Additional words were added by researchers only to assure readability. The participants’ expressions were then ordered according to the original temporal sequence of the life situations they described. The analysis of the data was informed by the methods of phenomenological psychology, which were used to elucidate the essential meanings of the parents’ lived experiences of their child (Davidson, 2003; Giorgi, 2009; Wertz, 1983, 2005, 2010). In addition, empirical trends in the data, such as the relative frequency of important parental experiences and the chronological ages of children, were noted and summarized when possible. The presentation in this report includes both phenomenological findings, (i.e., the essential structure and constituent meanings of parents’ experiences) and the empirical findings (accounts of related factual trends evident in the data). Analytic rigor was ensured via internal reviews by expert members of the research team. In the resulting presentation, participants have been de-identified. Narratives have been identified by the notation of M(other) and/or F(ather) with accompanying participant number (see Table 1).

## Results

The following sections describe parents’ lived experiences with their child, with reference to context, meaning, and life historical unfolding. The findings indicate a series of temporal structures. Four main temporal phases emerged from the data and are described in what follows. It was not possible to list the exact chronological duration of each because there were no definite boundaries between them. The age of the child when each phase began also varied considerably between parents, and some parents had not entered the latter phases.

In each of the four phases, the following three constituents were essential to parents in caring for their child: (a) Engaging in an open-ended process of parenting by learning to meet new and unfamiliar challenges; (b) Ensuring that the child’s basic needs are met; and (c) Finding an engaging niche with a sense of belonging for their child in the everyday milieu including social relationships, education/work, maintaining family values, etc. These aspects of parenting were present in the earliest period and continued to develop in later ones as described in what follows.

## Beginning the parenting journey: Reciprocal routines, budding care networks, and an open future

In the earliest period after the child’s birth, parents happily welcomed the newborn and embarked upon the journey of care by attempting to develop harmonious, synergistic routines. Most parents identified this early period as joyful and honored the child with pride.

When he was born there was a huge celebration and a lot of excitement (M10).

My daughter was so very cute and very good looking and on seeing her I forgot everything. All the negativity in me, it went away on seeing her (M1).

It was like picture postcard. He won about three “Healthy Baby Contests” (M6).

The immediacy and novelty of their new life with their son or daughter was predominately positive:

Her first birthday actually I took time off (M1). We celebrated in a very big way (F1). We called relatives and it was a big party and … We took photographs (M1).

The child’s behaviors were expected, ordinary, and normal. Although parenting was an inexact science, parents gained sufficient know-how to respond to their child. Caring practices were informed by prior experiences of parenting, advice from extended family, and basic learning-by-doing, trial-and-error approaches. Parents did not extensively contemplate the long-term future; however, the baby’s future was given with open possibilities.

“Autism” and troublesome behaviors beyond the scope of routine parenting had yet to appear. If significant medical problems of the child appeared, courses of action were clear, such as taking the child to a doctor for excessive vomiting. In essence, their child’s medical issues were problem-solving situations in a world that was familiar to them. The anticipation was that solutions provided by doctors would enable the family to return to normal routines. Although ASD had yet to emerge, the caregiving skills and social networks that parents were now developing would later become essential resources when disruptive and unexplainable behaviors began to appear. For instance, the child’s early medical problems led parents to form relationships with physicians and extended family as allies in childcare. The nature and quality of these early social networks shaped the course that parents’ care efforts eventually took with regard to the child’s later behavioral problems. Two parents (M5, M10) recounted their child’s numerous early medical problems, which required extensive travel for specialized healthcare. One parent described her village as “a place where you don’t even have access to good doctors.” She was greatly dissatisfied with the lack of available local support and thus grew accustomed to traveling afar for better care. Both M5 and M10 experienced professionals as generally insensitive to the family’s situation, leaving an early negative impression that would later color expectations of various service providers when, with the appearance of ASD, the quality of relationships with service providers became a crucial issue.

## Interruptions from the path: Child’s unexpected, incongruent, but seemingly temporary behaviors

Various unexpected behaviors of the child caught parents’ attention due to their disruption of the social cohesion of everyday life. These behaviors were incongruent with those of older siblings or same-aged children and parents’ own underlying views of expected childhood behavior. Other family members, however, normalized the incongruence by mentioning similar patterns in family history or cultural norms, for instance, concerning the typical age of speech onset (e.g., “your husband also used to also speak late”; “sometimes *Jeep Zodh aasa* [the tongue is thick]”). In this way, the discrepancies were ordinary and integrated into the prior experiential phase of parenting. Further examples of these behaviors of the child included: (a) Trouble falling asleep (M1); (b) Agitation in a restaurant during a family vacation with parents’ work colleagues (M6); (c) Crying continuously without stopping, and the parent not knowing why (M4); (d) Not sitting (F3, M10); (e) Flapping hands (M10); and (f) Crying so softly that it resembled a “cat’s meow” (M5).

The child’s unexpected, discrepant behaviors began affecting the everyday parent–child relationship, leading some parents to ponder their own caregiving efforts. However, some also noted adaptive aspects of the unexpected behaviors. For instance, one parent (M4) could not soothe her child’s crying, and two others (M1, F1) were surprised that their child did not seem very attached to them. In the first case, the parent could not understand why her soothing efforts weren’t working; however, in the second case, the parent felt that her daughter’s detached manner was actually a “blessing in disguise” because the child didn’t seem to miss her when the mother had to leave the house.

But one thing it was a bit strange that she didn’t seem to miss me very much sometimes. (M1)

Not that she used to dislike her or anything like that. (F1)

But that I took as a blessing in disguise. I thought that it’s good that she is not missing me. She was quite happy with her nanny and my mother-in-law. (M1)

In the parents’ understanding, these discrepant behaviors were not indicative of a larger or ongoing issue but instead were temporary. Although some parents began to doubt, their friends and family comforted them with reassurances that the child’s atypical behavior was just part of the ordinary process of child growth. Parents described that:

Nobody thought that there was anything amiss. (M6)

At 8 months I was comparing him to other children, and I felt they used to do a lot more, but it still didn’t … cross my mind that there is problem. (M10)

Even as parents’ perspectives on their child became increasingly divergent from reassuring others, they continued to believe that the disruptions would eventually pass. The parents continued to anticipate a process of change that would lead to their child occupying a familiar place in adult society. The more distant future remained open and potentially limitless.

When the term “autism” was introduced early in this phase, its meaning for parents remained relatively empty and without significant implications. Parents may have heard the word but did not apply it to their child due to a lack of familiarity or because their child seemed to function well in most areas of life.

That first time when we were told autism, we had taken it carelessly. We had never heard about it. (F2) And he was okay in other things (M2).

His only problem was that he was not communicating. He was not communicating but other things he was doing. He used to understand us if we told him anything. (F2)

“Autism” was relatively unknown. Perhaps it applied to others, but it was inconsequential with regard to their own child, because challenging behaviors were isolated, transient, and did not hold meaning concerning the child as a whole with implications for the future.

## Roadblocks along the way: Child’s continued growth in question, and parents’ search for solutions

When behaviors began to be seen as different from other children and as socially problematic beyond the immediate family, parents sensed that they were persisting and potentially dangerous for the child. One parent stated:

He used to throw this and that, he used to remove and throw a burning stick from the *chula* [open flame cooking stove], he would touch the hot iron, he would dip his hand in boiling tea, he used to throw the milk out. Sometimes we would starve and [lose] sleep for this reason. (M7)

The most alarming and transformative events that initiated the third phase involved unexpected and intrusive communication from other concerned persons. One parent (M10), for example, was verbally accosted by a doctor for “poor parenting” after the parent brought her son to the doctor for his flu-like symptoms. The doctor admonished the mother for not recognizing the implications of the child’s speaking, sitting, and walking difficulties, after which she began elaborating their significance for the future.

The way he disclosed it to me, it came as a total shock to me because he said, “Oh my God, he will be a vegetable for the rest of his life. What kind of mother are you, you don’t know how to take care of a child?” … I just did not know what to do. I was just totally in a state of shock. (M10)

Although parents became concerned about the child’s fate and eventual place in adult society, the long-term future was still a distant, though increasingly uncertain, horizon. Parents were concerned with the possibility that their son’s or daughter’s ways of being may preclude their capacity for self-care, social relationships, and education. Consequently, the behaviors identified by teachers and administrators at schools engendered the most concern among parents. Parents had viewed education as the gateway towards a brighter future in which their children would gradually overcome relative dependence on them. However, these growing questions threw that expectation into uncertainty. Though the parents were increasingly concerned, hopes still remained that the child’s problems would subside. One father, a cattle herdsman, recounted taking pleasure in positive gains after earlier concerns about his son’s performance in school:

Now if I go to give fodder to the cow, he goes first and gives them. He comes and mixes all fodder and gives them. He knows everything. Now he says that he wants two cows, one red and one black. So it feels good. (F3)

Before this phase, the children were seen as proceeding along a typical pace. However, when the child began entering the outside world, perspectives of normality/abnormality from medicine or media came to the fore. In other words, this phase was when “autism” began to take on determinate meaning for parents in reference to their own child. Comparisons with other children and experts’ views introduced the meaning of a persisting problem with implications for important areas of life (education, relationships), but their total implications for the future were not yet elaborated in concrete ways. Allopathic and alternative healers were consulted, including an Ayurvedic doctor who determined that a “nerve is getting choked up somewhere” (M4) and a *baba* (religious man) who concluded there was “a *shraap* [curse] and [a] need to perform a Ganesh *aarti* [religious service]” (M9).

Some parents continued to hold on to dreams such as the arrival of a medical advancement or cure, the hope of a desirable job in government for the child, or the wish of the child becoming an “Einstein” or “Beethoven.” These connotations of scientific/musical genius did not necessarily match parents’ own observations of the child but were consistent with other meanings of the diagnosis learned from media and professionals. A concurrent manuscript (Divan, Vajaratkar, Desai, Strik-Lievers, & Patel, in press) discusses parents’ interactions with professionals in more detail.

## Here, but not yet there: Addressing the present while looking towards the future

In this phase, the parents’ aim was to accept their child’s limited capacities, to expand them as much as is possible in relation to the given social realities, and to attempt to change those social realities so that they would become more welcoming for their child. It should be noted that not every parent in our study had fully reached this phase, such as the rural father who still assumed his child would be given a government job. The children in this phase were usually nearing their teenage years. The meaning of age here reflects normative parental beliefs about what the child should be doing at their current age with regard to learning, communicating, socializing, and so forth. As one parent discussed, when it became certain through persistent observations that her child was not performing in the classroom like his peers, it finally “dawned” on her that the child was not going to become a “typical” adult. Previous doubts concerning the child’s abilities have here become certainties. In this phase, parents began to give up the expectation that their child would reach the hoped for self-care, social, or vocational capacities of other adults.

Parents accepted and owned their child’s limitations as a consequence of seeing that their child remained different from other children in situations essential for becoming a fully functioning adult in the local setting. In this way, the label “autism” was filled in with meanings with implications for the child’s whole life—past and future. Parents were learning more about professional or scientific knowledge related to autism, and their own understandings of their child often changed as a result. For example, parents started to retrospectively reinterpret earlier perceptions as indicative of symptoms of ASD, and some reported their own indigenous care efforts to be consistent with professional therapy for autism.

Whenever we were in the restaurant, he used to get agitated. That was probably the first sign of socialization problem. (M6)

She was content to be with herself. That’s probably one of the red flags but at that time you do not realize; you do not know anything about autism. (M1)

In my intuition I taught her everything I could. And I was kind of doing ABA [applied behavior analysis] with her (M1). I did not know the term but I was doing ABA with her. She was looking outside the window and I was holding her head and literally turning her head; I was forcing her to make eye contact. (F1)

Even when the concept of autism was accepted as applicable to their child, most parents also attributed their child’s unusual differences to multiple, alternative, and often conflicting external causes, but remained unsure as to any final solution. Some general trends of explanation included: (a) genetics or chemical exposure (M1, a PhD scientist); (b) potential links to other children in the family with developmental issues, e.g., a “spastic” relative (M6); (c) mother not taking required medications while pregnant, or taking too many medications (F3, M10); and (d) stressful experiences during or after pregnancy.

As the children aged, parents came to assume a previously unexpected role as the one ultimately responsible for their son’s or daughter’s everyday needs. But what about the future? All parents in this fourth temporal phase deeply worried about what would happen to their child when the parents “are no longer,” that is, when the parents could no longer care for their children. One parent advocated for a social scale security system in India, whereas another worried:

Later on … who’ll take care [of him]? … He is independent but not independent enough to go out and all. (M5)

A major concern for parents became discovering their son’s and daughter’s place in the world and finding ways to help them occupy it. Some parents attempted to organize activities and find creative outlets where their child would belong and have an enjoyable present and future. One mother noted:

I don’t have expectations but I just want him to live a happy life. Enjoy every moment that we enjoy with him. Every moment of his has to be enjoyable and I don’t want any pressure on my son. (M5)

Parents also sought to maintain values and traditions like religion, spirituality, and art.

First he used to not mix with other children, but now he likes to play with them … He acted as Krishna [Hindu god] for his school program. That time he wore Krishna’s costume like *mukut* [crown], *murli* [flute]. (M7)

Given the difficulties of finding or establishing an alternative social milieu, patience became a virtue for many parents as they waited for their children to learn basic life skills, albeit in their own way: “I think we have to keep our patience. You can rush things with other kids but not with her. She will learn in due course” (M1). Another parent poignantly described: “He should be able to lead his own life; [he] should be able to write his own name, we don’t want him to be great but at least that much he should do” (M4).

Although “autism” took on a certain reality with implications for both the past and future, it is not necessarily a fully determinate fate or destiny, for open questions remain about what the child ultimately can become, what the parent can do, and how social realities can be changed to be more welcoming and accommodating to their sons and daughters.

## Discussion

This study conducted in Goa, India, has limits of generalizability because all participants had at least some contact with the health or educational system. The findings indicated that parents’ experiences of caring for their child with ASD involved three main constituents, which emerged through four temporal phases: learning to meet new and unfamiliar challenges as parents; caring for their child’s basic needs; and finding an engaging niche with a sense of belonging for their child in the everyday milieu. The four temporal phases were: Phase 1, a period of celebration and social cohesion; Phase 2, though parents began to perceive their child’s behaviors as incongruent with expected behaviors, the discrepancies were understood as temporary and viewed by family members as within the bounds of ordinary childhood behavior; Phase 3, parents’ observations in public situations, along with assessments by others, led to a qualitative shift in which parents began to perceive a persisting problem interfering with practical and social activities; and Phase 4, parents recognized that these persisting behavioral problems rendered the child’s future place in the social world uncertain. Parents grappled with developing their child’s capacities to meet existing practical opportunities in the local society, while attempting to reshape the social world to accommodate the abilities and limits of children like their own. With regard to diagnostic conceptualizations, neither “autism,” nor any conception of a “developmental disorder” was present in Phase 1, and in Phase 2, parents disregarded the autism diagnosis as they focused on what they perceived to be temporary deficits in functioning (e.g., poor communication). “Autism” took on meaning for parents beginning in Phase 3, as indicating persisting problems in social and educational settings, but future implications were not fully experienced until Phase 4.

The present study is consistent with other literature on ASD and developmental disabilities in India and elsewhere and extends its findings to Goa. Research has noted that family members explain early deficits in functioning as actually typical for children. For instance, the suggestion that “boys just speak late” is a consistent finding in the literature on India (Daley, 2004; Krishnamurthy, 2008). Daley (2004) also pointed out that most parents reported social difficulties first; evidence from our study similarly indicates that difficulties in social settings, such as school, initiated the entry into Phase 3 where parents perceived that there was a persisting problem. Recently, researchers have also found that children’s basic self-care needs and capacity to live in society were central to parenting children with disabilities in South Asia (Edwardraj et al., 2010; Maloni et al., 2010).

There is a striking convergence between our findings and a recent study from the USA (Altiere & von Kluge, 2009), which appeared while the present study was being conducted and was reviewed after our findings were written. Both studies report similar evidence concerning a relatively cohesive early parenting phase; a later period of questioning and searching for solutions; and “hindsight” reinterpretations. The studies, however, differ in their descriptions of the meaning of “autism” for parents. In the present study, autism was not received by parents as a fixed entity and clear biomedical diagnosis. Instead, the idea and term were ambiguously perceived. Parents had initially never heard of the term autism, and subsequently disregarded it despite noticing some disruptive behaviors. At a later phase, parents understood autism as indicating an open-ended future. Both studies described this open-ended future, which, as Altiere and von Kluge pointed out, was characterized in part by patience, joy, social advocacy, and fighting for acceptance.

Research from the West has also noted a central paradox that parents of children with autism and other disabilities encounter, specifically of accepting their children as they are while also hoping for change by pursuing solutions to their difficulties (Avdi, Griffin, & Brough, 2000a; Landsman, 2003; Larson, 1998). Larson described the “embrace of paradox” in the face of an indeterminate future as follows:

the management of the internal tension of opposing forces between loving the child as he or she was and wanting to erase the disability, between dealing with the incurability while pursuing solutions, and between maintaining hopefulness for the child’s future while being given negative information and battling their own fears. (1998, p. 873)

This paradox was found to be central for Goan parents in Phase 4. Further, the present study extends the notion of paradox to include parents’ acceptance/change not only of the child but also of *the social world*. On the one hand, if parents do not accept the social reality and the requirements for participation in it, they cannot help their child adapt to it. On the other hand, parents cannot simply accept the social reality, because it is not entirely suitable or welcoming to their child—so they also endeavor to change the social world. In other words, there was a double paradox of acceptance/change of both the child *and* the surrounding environment. Parents embraced this paradoxical attitude towards the social world by accepting but changing the social reality. For instance, a group of mothers who helped their children adapt to the current social and practical realities in the local setting also worked with community members and professional mentors to advocate for more vocational opportunities, residential facilities, and a large-scale social security system. Grinker (2007) described similar instances of parents creating social change in India, such as the life story of Merry Barua, a mother of a child with ASD and subsequent founder of a national autism action network, advocating for relevant services and government recognition. These instances of parent empowerment in India resonate around the world with autism and disability advocates, such as those discussed by Grinker (2007), Shaked (2005), and others (Daley, 2004; Gray, 2001; Luong, Yoder, & Canham, 2009; Skinner & Weisner, 2007; Woodgate, Ateah, & Secco, 2008). Autism advocacy is also exemplified by Temple Grandin’s (2006) extensive discussion of “developing autistic talents” and focusing on strengths and opportunities over limitations alone.

Previous literature has called for more in-depth, cultural research on parents’ subjective processes (Daley, 2002, 2004; Kishore & Basu, 2011). Past research on ASD has demonstrated wide variations in the ways parents interpret their child’s early behavioral discrepancies, including consistent nonbiomedical interpretations, with an accompanying time lag between recognition of these discrepancies and professional help-seeking/diagnosis for many parents (Daley, 2004; Kishore & Basu, 2011). The period when parents initially perceive disruptive, unexpected behaviors corresponds with Phase 2 found in our study. Parents here perceived temporary deficits in functioning, but these were not viewed as part of a larger problem or as requiring intensive or extensive professional help. Even when the child was given a diagnosis of autism in Phase 2, parents disregarded it as they focused on the particular discrepant behaviors. These behaviors were only later seen by parents as persisting problems and, in Phase 4, as possibly chronic or as early symptoms/indicators of autism upon retrospection to earlier times.

Although the Indian literature has started to broach this issue, it has not sufficiently distinguished early parental perceptions from their later interpretations or the interpretations of professionals. For instance, early parental perceptions have been described in the literature as “initial symptom recognition” (Daley, 2004, p. 1327) and “early indicators of autism” (Kishore & Basu, 2011, p. 157), which are parents’ *retrospective* reinterpretations of their child’s early behaviors in later phases. Parents originally did not perceive a behavioral problem of the magnitude of symptom/disorder. More accurately reflective of parents’ initial perceptions are such descriptive phrases as “some aspect of development … not proceeding as expected” (Daley, 2004, p. 1327) and “early concerns” and “atypicalities” (Kishore & Basu, 2011, p. 162). We propose that this is an important distinction because it may be difficult to fully engage parents without addressing their concerns in ways that are meaningful to them in their particular experiential phase.

### Practice implications

The importance of direct parental involvement in the creation and provision of any services for special-needs children in India has been previously noted (e.g., Gupta & Singhal, 2005; Kalyanpur, 1996). Though professional perspectives differ from those of parents at various points, the present study indicates that professional practices can be related to the essential concerns of parents, and a common ground of understanding and shared purpose is possible.

With regard to the earliest phases, it is important for policymakers to work with communities to help build family relationships with local care systems, remaining respectful of their ways of life and points of view. In the present study, general care networks that supported parents prior to their noticing any serious behavioral discrepancies became relevant and useful when parents perceived a persisting problem. One mother who developed positive relationships with local care providers in the first two phases became empowered by them later when serious problems emerged. Another mother, however, not only lacked supportive service relationships in the first two phases, but had limited local availabilities for care altogether and needed to travel long distances for other options (see also Daley, 2004, for discussion of geographic/socioeconomic issues in India). For families facing these situations, if caring relationships are developed earlier within the local care system, then parents may feel more empowered and receive immediate care when serious problems emerge.

Regarding Phase 2, though parents view discrepant behaviors as temporary and not a lasting problem for the child, there is an opportunity for communication between family and external helpers. Specifically, the child’s disruptive, unexpected behaviors can pose problems for parents, even if they do not see those problems as requiring extensive professional attention. Here, parents may be receptive to basic advice that addresses their child’s challenging behaviors, such as difficulty falling asleep or uncontrollable crying, which were beginning to exceed parents’ own management capacities in this study. One potentially relevant kind of community resource would be a local support network comprised of parents or caregivers of children with or without ASD as a source of relational support and a resource for “best parenting practices.” Several other researchers have noted the value of parent networking for childhood disabilities in South Asia (Goldbart & Mukherjee, 2001; Gupta & Singhal, 2005; Maloni et al., 2010; Mirza, Tareen, Davidson, & Rahman, 2009; Pal & Chaudhury, 1998; Russell, John, & Lakshmanan, 1999).

Professionals might recommend early intervention to parents in Phase 2 or earlier, when they have not yet experienced a problem that requires it. This gap presents a dilemma, and further research is warranted. Parents’ major pathway to realizing their child’s long-term needs took place in social situations where they were able to compare their child to other children. A parent network could offer parents in Phase 2 a space to process how their child interacts with others, potentially occasioning an earlier perception of a persisting problem. Such a parent network could be particularly valuable for parents in India who are socially isolated, lack financial resources, and posses less knowledge of vocational requirements (see Maloni et al., 2010). For example, the cattle herder in this study did not appear to have means of accessing resources comparable to those of urban, highly educated parents when he needed them. As he succinctly put it: “Poor people like us find it difficult.” At the community level, families in Goa were aided by parent networks formed by a few key parent leaders with the help of professional mentors.

The present study provides evidence of a conflict of beliefs initiated by professional diagnosis in Phase 1 or 2. Professional views may clash with the interpretations of parents and extended family members. This possibility of conflicting viewpoints echoes previous research, which has demonstrated incongruity between parents’ perceptions of their child and scientific or biomedical interpretations offered by experts (Avdi et al., 2000b; Feinstein, 2008; Gray, 1995*,* 2001; Landsman, 2003; Mandell & Novak, 2005; Shaked*,* 2005). In the present study, only after extended observations of their children in relation to other children in social situations did parents gain some realization of the concrete implications of their child’s difficulties (Phase 3). But these realizations took place after what we have called Phase 2, in which the parent viewed the unexpected, incongruent behaviors or deficits as temporary. Scholars have raised cautions about applying Western constructs of abnormality, disease, and treatment to novel populations or cultures (Gone, 2004; Kalyanpur, 1996; Shweder, 2008; Watters, 2010). Our research suggests that without opportunities that will help parents concretely realize and explore their child’s capacities in social situations, diagnostic constructs are likely to have a largely indeterminate meaning that remains at a distance from parents’ and extended families’ practical hopes and activities. This distance limits the potential for parent–provider collaborations.

Beginning in Phase 3 and especially Phase 4, when parents contemplate the future implications of their children’s differences from others, their immediate concern is for alternative social situations that will offer their children opportunities to fulfill their potentials. Here arises the interplay of both the acceptance and reshaping of reality, possibly with the help of others. Professionals—medical, educational, or alternative—could play a key role in understanding this paradox, similar to what Larson (1998) called being “embracers of paradox” (1998, p. 871). Though usually trained primarily to change the child’s behaviors, professionals could also offer knowledge and awareness of social opportunities, and of how to creatively reshape them, as another primary role in serving families. Social action possibilities include developing more diversified paths for adult living for people of all walks of life. There are examples in our study of parents searching for alternative adult livelihoods, such as basic manufacturing and the arts. More opportunities can be created locally, ranging from sustainable jobs to a restructuring of society’s safety nets for differently abled children and adults. To be sure, scenarios where the child adapts to local opportunities, has their needs met, and engages in the tasks required of adults in the local milieu are possible. For example, a key informant (healthcare worker) mentioned witnessing a child with potentially diagnosable intellectual disabilities functioning reasonably well in the shepherding work expected by the child’s family. Development of these diversified pathways is a locally negotiated process that could be enhanced by the exploration and creativity of all interested parties mentioned in this study (see also Grinker, 2007).

### Cultural/transcultural

The study began with an intent to discover how parents in Goa indigenously understood and cared for their child with ASD. Our study shows that research is needed to shed light on both the local and potentially universal (i.e., highly general) aspects of parental care of a child with ASD. In Goa, examples of the three-part structure—learning to meet the new and unfamiliar, caring for their child’s basic needs, and finding an engaging niche with a sense of belonging for their child in the everyday milieu—included the joy of seeing one’s son identify with the family’s agrarian way of life, the danger of a child throwing a stick from an open flame *chula* cooking apparatus, and the importance of performing in a Hindu play. Research elsewhere suggests that the four-part developmental sequence and three-part structure of parenting may be found in other cultures and with childhood problems other than ASD. Altiere and von Kluge (2009) described distinct phases including the child developing “normally,” parents’ questioning then searching for solutions, and personal/family growth. Similarly, Shaked’s (2005) study of autism in ultraorthodox Israel demonstrated how parents struggled to reintegrate their child into the religious community that they held so dear by both accepting and challenging social norms (i.e., learning to meet the new and finding engagement and belonging). Further, Grinker’s (2007) poignant narratives of parents of children with ASD included the story of a New York parent who devoted her life to creating alternative vocational and recreational centers (i.e., engagement and belonging) and lifelong housing (i.e., care for basic needs) for older persons with autism. Many aspects of parents’ concerns for their child in the present study appear to be similar to those of parents much more generally, but the manifestations of each caring practice are flavored by the local cultural customs and dynamics. Those local flavors are, in turn, influenced by parenting traditions, availabilities for childcare, and the opportunities for engagement and belonging in the family and community. More research is needed in order to further clarify potentially universal features as well as the many richly diverse local manifestations throughout the world.

## Limitations and conclusion

The present study has several limitations. The sample was limited to parents who have had contact with professional care centers and whose child attended various services regularly. Families who never made contact with professionals were not included; the sample thus offered a picture of only a limited segment of local experiences. Also, the study relied on interviews with parents, whereas direct observations of family life were not systematically conducted. Consequently, the current study could not do full justice to the complex everyday experiences of families. The present study in Goa revealed similarities to other areas in India; however, more research from other regions in this vastly diverse country would be needed to further confirm and extend the conclusions of this paper. Further research is particularly needed for examining instances of parents and professionals fruitfully collaborating on the paradox of child acceptance–social acceptance/child change–social change.

This investigation of ASD in India reveals a rapidly changing and dynamic landscape. DSM and ICD diagnostic criteria are increasingly utilized by professionals in India (Daley & Sigman, 2002). The descriptive approach of this study and focus on meaning provided insights into parents’ own perspectives and their discovery of the concept of autism in the midst of these historical developments. Parents’ experiences of their child diagnosed with ASD were found to be intimately tied in with fundamental human concerns: that a child is cared for, belongs in the social world, engages in practical activities, and finds enjoyment and contentment in life. These findings imply that helping families meet these needs locally can go a long way towards supporting parents and children in their life journeys.

## Appendix. Interview guide

### In-depth interview guide for parents

#### Icebreaker

- Let’s begin with telling me about your family
- How many people stay in this house (with you)?
- What are the children’s names? How old are they?

#### Interview

- Tell me more about your children.
- Probe: Pregnancy planned/unplanned? Experience of pregnancy. Childbirth normal/complicated? Personal emotions, first 3 years or until the parents first suspected/were told that XXX may be different
- As XXX grew up what was life like in the early days?
- Probe: When did you notice anything different about the way she/he was growing? Compared to whom? For what reasons? Did you call it anything in particular? What did you do? If different then how did you manage? Challenges in looking after XXX? What helped? What didn’t?
- As XXX grew how did people respond to him?
- Probe: Were there any behaviors which upset people, family members, siblings, community?
- Is there any advice that was given to you? What helped? What did not? What has changed since XXX was born?
- What did you think was the cause of XXX differences?
- Probe: Did others have a different opinion? Members of extended family? Siblings? Neighbors? Community? Doctors? If cause changed since early days, probe what made this change happen.
- Probe: How the understanding changed over time? For what reason did it change? Track this process through to the current understanding.

#### Help seeking

- What made you first think you should turn to somebody outside the family for help? (behavior/worry)
- Who did you go to first?
- Probe: Draw a timeline of all persons visited. Probe for each: reasons for consulting this person. What did they tell you? What did you think of the advice (good/bad)? What was done after each interaction? Ask specific: priest, other help. What are you continuing with now?
- Are you satisfied with what was told to you was the problem? How has this influenced your understanding of your child’s behaviors?

#### Impact/stigma/discrimination

- What is life like with XXX now? Are there any changes that you have made to your day-to- day life?
- Probe: Siblings? Husband/wife? Emotional/financial implications/social/work?
- Probe: Is there anyone you would not tell? Why?
- Are there any places or occasions that you don’t want to go to? What are the reasons? What do you do for XXX’s birthday? (Compare with other siblings).
- Has your relationship with anyone changed because of XXX; how/why?
- Is there any local way/words that people use to refer to XXX? Ways in which people behave towards him/her?
- Probe: What do their terms represent to you? How do you feel about people using such terms?
- Since the diagnosis has anyone’s behavior changed towards you as a family? How do you handle it?
- Have you had any unpleasant experiences related to how you or your child was treated by others? Can you describe a recent experience/worst experience? What happened? What did you do? How did you feel about this? And your child? How has this affected your life?
- Enjoyable moments?

#### Support

- What support do you have from outside or in the family?
- Probe: Do you know any other family with children with disabilities? Do you meet up with them? Religious agencies? Other sources? Helpful?

#### Current management and services

- What has helped you look after XXX? How do you manage? Who helps you? In what ways? What is most helpful?
- What treatment is he/she on now?
- Probe: What care is XXX receiving now?
- What is missing?
- What is your greatest hope?
- What needs to be done to get there?
- What are your expectations? (Your own/family, services provided to you?)
